# The expression profile of Dopamine D2 receptor, MGMT and VEGF in different histological subtypes of pituitary adenomas: a study of 197 cases and indications for the medical therapy

**DOI:** 10.1186/s13046-014-0056-y

**Published:** 2014-07-16

**Authors:** Youwei Wang, Junyang Li, Mamatemin Tohti, Yuebing Hu, Sheng Wang, Wanchun Li, Zhenfeng Lu, Chiyuan Ma

**Affiliations:** 1 Department of Neurosurgery, Jinling Hospital, School of Medicine, Nanjing University, 305 East Zhongshan Road, Nanjing 210002, China; 2 Department of Pathology, Jinling Hospital, School of Medicine, Nanjing University, 305 East Zhongshan Road, Nanjing 210002, China; 3 Department of Neurosurgery, Yangzhou No.1 People’s Hospital, The Second Clinical School of Yangzhou University, 368 Hanjiang Road, Yangzhou 225012, China

**Keywords:** Dopamine D2 receptors, MGMT, VEGF, Dopamine agonists, Temozolomide, Bevacizumab

## Abstract

**Background:**

To study the expression of D2R, MGMT and VEGF for clinical significance in pituitary adenomas, and to predict the potential curative medical therapy of dopamine agonists, temozolomide and bevacizumab on pituitary adenomas.

**Methods:**

Immunohistochemistry and western blot were performed to detect the expression of expression of D2R, MGMT and VEGF in pituitary adenoma tissue samples. The ratio of high expression of D2R, MGMT or VEGF in different subtypes of PA was compared by the use of chi-squared tests. The relationships between D2R, MGMT and VEGF expression were assessed by the Spearman rank correlation test. The association between their expression and clinical parameters was analyzed using a chi-squared test, or Fisher's exact probability test when appropriate.

**Results:**

The data showed that in 197 different histological subtypes of pituitary adenomas (PAs), 64.9% of them were D2R high expression, 86.3% were MGMT low expression and 58.9% were VEGF high expression. D2R high expression existed more frequently in PRL- and GH- secreting PAs. MGMT low expression existed in all PA subtypes. VEGF high expression existed more frequently in PRL, ACTH, FSH secreting and non-functioning PAs. The data of western blot also support the results. Spearman's rank correlation analysis showed that expression of MGMT was positively associated with D2R (r = 0.154, P = 0.031) and VEGF (r = 0.161, P = 0.024) in PAs, but no correlation was showed between D2R and VEGF expression (r = −0.025, P = 0.725 > 0.05). The association between their expression and clinical parameters was analyzed using a chi-squared test, or Fisher's exact probability test when appropriate, but the result showed no significant association.

**Conclusions:**

PRL-and GH-secreting PAs exist high expression of D2R, responding to dopamine agonists; Most PAs exist low expression of MGMT and high expression of VEGF, TMZ or bevacizumab treatment could be applied under the premise of indications.

## Background

Pituitary adenomas (PAs) account for about 15% of intracranial tumors. Although PAs are mostly benign lesions, about 30-55% of them are confirmed to locally invasive, and some of them infiltrate dura, bone and sinuses, are designated highly aggressive [[1],[2]]. The conventional treatment of large pituitary adenomas consists of surgery, and radiotherapy when it is hard to achieve total resection. The use of additional radiotherapy is limited by the risk of radiation necrosis of surrounding structures. Thus, medication treatment, although unlikely to be curative immediately, might lead to certain clinically therapeutic effect, as a useful supplement [[3]].

Currently, first-line clinical medication for PAs generally consists of dopamine agonists (DAs), somatostatin analogs (SSAs) or combinations [[4]]. Recently, some routine chemotherapeutics such as Temozolomide (TMZ) and Bevacizumab have been carefully studied to treat PAs and considered to be potential for aggressive PAs’ medical therapy [[5]-[8]]. DAs were widely used for the treatment of prolactinomas and some somatotropinomas, and the responsiveness depends on the expression of dopamine D2 receptors (D2R) on tumor cells. Abnormal expression of D2R in prolactinoma was considered to confer resistance to DA treatment. Fadul et al. [[7]] first reported two cases of pituitary carcinoma received TMZ treatment, concluding that TMZ may be effective in treating pituitary carcinomas. After that, more and more studies demonstrated the inspiring therapeutic effect of TMZ on pituitary carcinomas and aggressive PAs. As a DNA repairase, O6-methylguanine DNA methyltransferase (MGMT) confers chemoresistance to TMZ [[9]]. Thus, tumors with low expression of MGMT are usually sensitive to TMZ. Bevacizumab is a monoclonal antibody which has been approved by USA FDA to treat colorectal cancer, non-small-cell lung carcinoma, breast cancer, renal carcinoma and recurrent glioma [[10]]. It blocks vascular endothelial growth factor (VEGF) binding to its receptor [[11]]. Experimental and clinical studies have demonstrated that anti-VEGF therapy may be effective in pituitary carcinoma and aggressive PAs.

To investigate D2R, MGMT and VEGF expression profile in PAs, and to evaluate the status of the drug targets of DAs, TMZ and Bevacizumab for PA medical therapy, herein, we performed the immunohistochemical staining in 197 cases of different subtypes of PAs.

## Methods

### Patients and tissues

One hundred and ninety seven pituitary adenomas (PAs) of different histological subtypes were selected randomly from patients operated between 2009 and 2011 in the Department of neurosurgery, Jinling Hospital, School of Medicine, Nanjing University. All PA tumor tissues were formalin-fixed and paraffinembedded resected and then pathologically diagnosed, including 28 PRL-secreting adenomas, 20 GH-secreting adenomas, 27 ACTH-secreting adenomas, 15 TSH-secreting adenomas, 37 FSH-secreting adenomas and 70 non-functioning adenomas.

### Immunohistochemical staining

A streptavidin-peroxidase (SP) method was used for immunostaining. Briefly, slides were deparaffinized with xylene three times (each for 5–10 min), dehydrated three times in a gradient series of ethanol (100%, 95%, and 75%), and rinsed with PBS. Each slide was treated with 3% H2O2 for 15 min to quench endogenous peroxidase activity. Nonspecific bindings were blocked by treating slides with normal goat serum for 20 min. Slides were first incubated with rabbit polyclonal anti-D2R (Abcam, Shanghai, China; 1:50), mouse monoclonal anti-MGMT (Abcam, Shanghai, China; 1:50) or mouse monoclonal anti-VEGF (Abcam, Shanghai, China; 1:50) overnight at 4°C, and then rinsed twice with PBS. Slides were then incubated with a secondary antibody for 15 min at 37°C followed by treatment with streptavidin–peroxidase reagent for 15 min, and rinsed twice with PBS. The slides were visualized with 3,3’-diaminobenzidine (DAB) for 3 min, counterstained with haematoxylin, and mounted for microscopy.

### Evaluation of staining

The slides were evaluated by two separate investigators under a light microscope (Dr. Wanchun Li and Dr. Zhenfeng Lu). Staining intensity was scored as 0 (negative), 1 (weak), 2 (medium), and 3 (strong). Extent of staining was scored as 0 (0%), 1 (1–25%), 2 (26-50%), 3 (51-75%), and 4 (76-100%) according to the percentages of the positive staining areas in relation to the whole carcinoma area. The sum of the intensity score and extent score was used as the final staining score (0–7). Tumors having a final staining score of >2 were considered to be positive, score of 2–3 were considered as low expression and score of >3 were high expression.

### Western blot

For western blot analysis, the lysates were separated by SDS-PAGE followed by transferring to an Immobilon-P Transfer membrane (Millipore Corporation, Bedford, MA, USA). Membranes were probed with primary antibodies followed by incubation with secondary antibody. Proteins were visualized with chemiluminescence luminol reagents (Beyotime Institute of Biotechnology, Shanghai, China).

### Statistical analysis

Statistical analysis was performed using SPSS 16.0 (SPSS Chicago, IL, USA). The ratio of high expression of D2R, MGMT or VEGF in different subtypes of PA was compared by the use of chi-squared tests. The relationships between D2R, MGMT and VEGF expression were assessed by the Spearman rank correlation test. The association between their expression and clinical parameters was analyzed using a chi-squared test, or Fisher's exact probability test when appropriate. P < 0.05 was considered to be statistically significant.

## Results

### Expression of D2R, MGMT or VEGF in PA tissues

**Figure 1:**
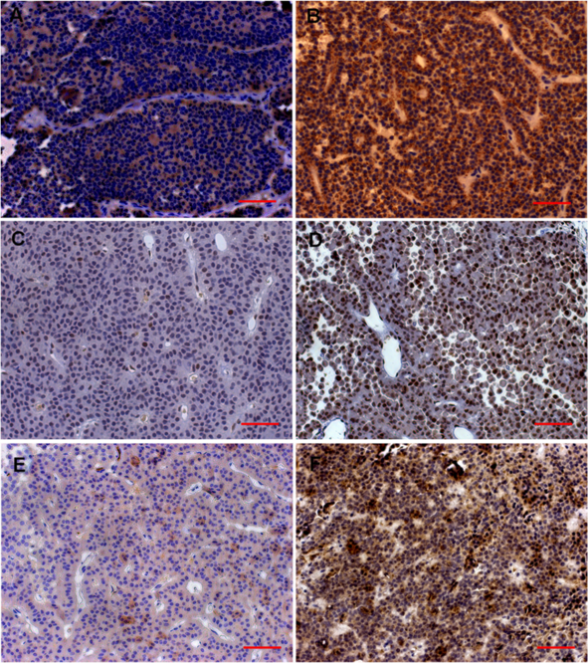
**Expression of D2R, MGMT and VEGF in PAs. (A, B)**: D2R low **(A)** and high **(B)** expression. **(C, D)**: MGMT low **(C)** and high **(D)** expression. **(E, F)**: VEGF low **(E)** and high **(F)** expression. Bar = 50 μm.

**Figure 2:**
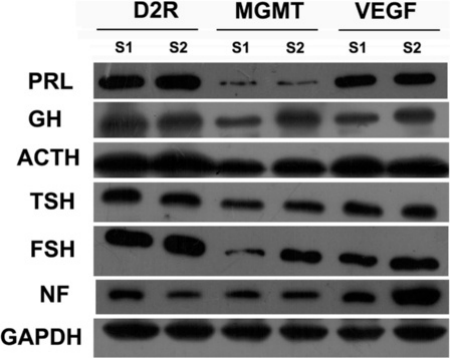
**The expression of D2R, MGMT and VEGF in different PAs subtypes by detected using western blot.** PRL: PRL-secreting PAs; GH: GH-secreting PAs; ACTH: ACTH-secreting PAs; TSH: TSH-secreting PAs; FSH: FSH-secreting PAs; NF: Non-functioning PAs. GAPDH served as loading control. S1 = Sample 1; S2 = Sample 2.

The location of D2R and VEGF in the nuclei and cytoplasm, and of MGMT in the nuclei was considered for scoring (Figure 1A–F). The positive expression of D2R was detected in 194 tissues, of MGMT was in all tissues and of VEGF was in 190 tissues. The proportions of cases showing low (score of ≤3) or high (score of >3) expression levels for D2R, MGMT and VEGF in different subtypes of PA were shown in Table 1. 64.9% of 197 PAs were D2R high expression, 86.3% of them were MGMT low expression and 58.9% of them were VEGF high expression. The ratio of high expression of D2R or MGMT is significantly different in PA subtypes (For D2R: χ^2^ = 44.844, P < 0.001; For MGMT: χ^2^ = 13.210, P = 0.021), but for VEGF, there is no significance (χ^2^ = 9.003, P = 0.109). D2R high expression existed more frequently in PRL, GH, ACTH, TSH and FSH secreting PAs. MGMT low expression existed in all PA subtypes. VEGF high expression existed more frequently in PRL, ACTH, FSH secreting and non-functioning PA. The data of western blot supported and confirmed these results (Figure 2).

**Table 1:** Expression profile of D2R, MGMT and VEGF in different subtypes of PA

**PA subtypes**	**No. of patients**	**D2R**		**MGMT**		**VEGF**	
		**Low**	**High**	**Low**	**High**	**Low**	**High**
PRL	28	2	26	24	4	11	17
GH	20	2	18	18	2	11	9
ACTH	27	9	18	22	5	13	14
TSH	15	6	9	14	1	8	7
FSH	37	6	31	26	11	8	29
NF	70	44	26	66	4	30	40
Total	197	69	128	170	27	81	116

NF, Non-functioning; Low, low expression (score of ≤3); High, high expression (score of >3).

### Relationships between D2R, MGMT and VEGF expression in correlation analysis

Spearman's rank correlation analysis showed that MGMT expression was positively associated with D2R expression (r = 0.154, P = 0.031) and with VEGF expression (r = 0.161, P = 0.024) in PA, but D2R expression did not show a correlation with VEGF expression (r = −0.025, P = 0.725 > 0.05).

### Association of D2R, MGMT and VEGF expression with clinical features of PAs

In these 197 cases, 106 of them were male and 91 were female; 64 of them were defined as invasive PAs, and others were non-invasive (according to Knosp’s classification [[12]]); 16 of them were recurrent PA, and the others were primary; 16 of them were microadenoma (diameter ≤ 10 mm), and the others were macroadenoma (diameter > 10 mm); 159 of the PAs were tender in tumor tissues, and the others were tenacious; Only 8 patients have taken bromocriptine orally. The associations between clinical variables and D2R, MGMT and VEGF expression are shown in Table 2. However, there was no significant association between D2R, MGMT or VEGF expression and clinical features, including patient sex, tumor growth pattern, tumor recurrence, tumor size, tumor tissue texture and bromocriptine application (P > 0.05). This indicated that despite the variety of PA clinical features, the expression of D2R, MGMT and VEGF are definite in PAs.

**Table 2:** Association of D2R, MGMT and VEGF expression with clinicopathological characteristics from patients with PA

**Parameters**	**No. of patients**	**D2R**		**P**	**MGMT**		**P**	**VEGF**		**P**
		**Low**	**High**		**Low**	**High**		**Low**	**High**	
Cases	197	69	128		170	27		81	116	
Gender				0.736			0.826			0.646
Male	106	36	70		92	14		42	64	
Female	91	33	58		78	13		39	52	
Aggressive				0.410			0.220			0.602
Yes	64	25	39		58	6		28	36	
No	133	44	89		112	21		53	80	
Recurrence				0.741			0.096			0.199
Yes	16	5	11		16	0		9	7	
No	181	64	117		154	27		72	109	
Tumor size				0.829			0.884			0.823
≤10 mm	16	6	10		14	2		7	9	
>10 mm	181	63	118		156	25		74	107	
Tumor texture				0.309			0.913			0.090
Tender	159	53	106		137	22		70	89	
Tenacious	38	16	22		33	5		11	27	
Bromocriptine				0.096			0.919			0.344
Yes	8	5	3		7	1		2	6	
No	189	64	125		163	26		79	110	

Low, low expression (score of ≤3); High, high expression (score of >3).

## Discussion

Dopamine D2 receptor is expressed in the anterior and intermediate lobes of the pituitary gland. The response to dopamine agonists is related to the activity of the D2 receptor which belongs to the family of G proteincoupled receptors and acts through AMP cyclase enzyme inhibition [[13]]. de Bruin et al. demonstrated that D2 receptor expressed in more than 75% of the cell population in normal human pituitary, indicating that D2 receptors are not expressed only in lactotrophs and melanotrophs, which represent no more than 30% of the entire cell population of the normal pituitary gland [[14]]. In PRL secreting pituitary tumors, the high espression level of D2 receptor explains the good therapeutic response to dopamine agonists, which induces tumor shrinkage. In present study, we investigated the expression of D2R in 197 cases of PAs and found that approximately 92.9% of prolactinomas and 90% of somatotropinomas are high expression of D2R, indicating potential good drug-sensitivity for dopamine agonists (DAs). Previous clinical studies revealed that cabergoline and bromocriptine can normalize serum PRL levels in more than 80% of prolactinomas patients [[15],[16]] and have a good effect in somatotropinoma patients [[17]], which consistent with our data from immunostaining analysis. Our data also showed 83.8% of FSH-secreting PAs and 66.7% of ACTH-secreting PAs are high expression of D2R, which is supported by several other reported studies, although clinical studies showed a long-term cure of 48% in cabergoline treated ACTH-secreting PAs [[18]-[20]]. Only 37.1% of non-functioning (NF) PAs highly expressed D2R according to our data, consistenting with the report by Colao et al. that the cumulative evidence for NF PAs shrinkage after DA therapy is 27.6% [[21]].

MGMT is a DNA repair protein that counteracts the effect of TMZ which is used for malignant glioma standard treatment. Recently, more and more studies revealed the therapeutic effect of TMZ on PAs, especially on aggressive PAs and pituitary carcinomas. MGMT expression as assessed by immunohistochemistry may predict response to temozolomide therapy in patients with aggressive pituitary tumors [[7],[22]]. McCormack group demonstrated that low MGMT expression and MGMT promoter methylation were found in the pituitary tumor of the patient who responded to TMZ, high MGMT expression was seen in the patient demonstrating a poor response to TMZ [[23]]. They reported the results that eleven out of 88 PA samples (13%) had low MGMT expression, and that prolactinomas were more likely to have low MGMT expression compared with other pituitary tumor subtypes. Herein, in this study we detected 170 out of 197 PAs (86.3%) existing MGMT expression lower than 50% (<50%) which was considered to be low MGMT expression. This data was higher than that form reported clinical studies in TMZ treated functioning PA, non-functioning PA and pituitary carcinoma with the remission rate of 75%, 55% and 72% respectively, which can be explained by Bush’s study that not all MGMT low expression PA respond to TMZ although medical therapy with TMZ can be helpful in the management of life-threatening PAs that have failed to respond to conventional treatments [[24]]. Our results showed low MGMT expression (<50%) in 85.7% of PRL-secreting PAs, 90% of GH-secreting PAs, 81.5% of ACTH-secreting PAs, 93.3% of TSH-secreting PAs, 70.3% of FSH-secreting PAs and 94.3% of non-functioning PAs, predicting almost all subtypes of PAs are suitable for TMZ therapy, although only fewer curative cases were separately reported [[25],[26]]. Further large scale clinical trials are necessary.

VEGF is a key mediator of endothelial cell proliferation, angiogenesis and vascular permeability. It plays a pivotal role in the genesis and progression of solid tumors. Onofri et al. analyzed VEGF protein expression in 39 cases of PAs, found only 5 cases (13%) were VEGF negative [[8]]. Lloyd et al. examined 148 human pituitary adenomas for VEGF protein expression by immunohistochemistry, and showed positive staining in all groups with stronger staining in GH, ACTH, TSH, and gonadotroph adenomas and in pituitary carcinomas [[27]]. Our study detected 190 positive VEGF expression cases in 197 PAs and 58.9% of them are in high expression level, including 60.7% of PRL-secreting PAs, 78.4% FSH-secreting PAs, 51.9% ACTH-secreting PAs and 57.1% non-functioning PAs. Niveiro et al. investigated VEGF expression in 60 human pituitary adenomas, and found that low expression of VEGF was seen predominantly in prolactin cell adenomas, and high in non-functioning adenomas, which is different from our data that 60.7% of prolactin cell adenomas verses 57.1% non-functioning adenomas [[11]]. Moreover, VEGF was considered also involved in conventional medical therapy for PAs. Octreotide was reported to down-regulate VEGF expression to achieve antiangiogenic effects on PAs [[28]]. Gagliano et al. demonstrated that cabergoline reduces cell viability in non-functioning pituitary adenomas by inhibiting VEGF secretion, of which the modulation might mediate the effects of DA agonists on cell proliferation in non-functioning adenoma [[29]]. Interestingly, in present study, we did spearman’s rank correlation analysis and found that D2R expression did not show a correlation with VEGF expression. Although it is prospective to treat PAs by anti-VEGF, up to now, only one case of PA has been reported to be cured by bevacizumab [[6]]. The mechanisms of VEGF in PA genesis and progression are still unclear. More studies are needed to investigate the effects of anti-VEGF therapy on PA patients.

To confirm the results, we also detected the expression of D2R, MGMT and VEGF by using western blot. The data supported the results of immunohistochemical staining. Two samples were selected for each PAs subtype. The positive expression of western blot indicated the immunohistochemical staining is available, and the thickness differences of the blot band revealed the expression level differences in separate sample.

Moreover, by spearman’s rank correlation analysis, we found that MGMT expression was positively associated with D2R and VEGF expression in PAs. As far as we know, it is the first time to report the association of D2R and MGMT expression which is positive. Only one report by Moshkin et al. has ever mentioned the association of MGMT and VEGF expression in PA. They demonstrated a progressive regrowth and malignant transformation of a silent subtype 2 pituitary corticotroph adenoma, with significant VEGF and MGMT immunopositivity [[30]]. The association between VEGF and MGMT expression in PAs need further investigations, as well as D2R and MGMT expression.

In addition, we analyzed the association of D2R, MGMT and VEGF expression with clinical features of PAs, but no association was found. This indicated that their expression was not affected by the differences of clinical features, and that the medical therapy can be applied in any patient in need.

In conclusion, in this study we demonstrated the expression of D2R, MGMT and VEGF in 197 different histological subtypes of pituitary adenomas, and analyzed the relationships between D2R, MGMT and VEGF expression and the association of D2R, MGMT and VEGF expression with PA clinical features including patient sex, tumor growth pattern, tumor recurrence, tumor size, tumor tissue texture and bromocriptine application. Our data revealed that PRL-and GH-secreting PAs exist high expression of D2R, responding to dopamine agonists; Most PAs exist low expression of MGMT and high expression of VEGF, TMZ or bevacizumab treatment could be applied under the premise of indications.

## Abbreviations

PA: Pituitary adenoma

D2R: Dopamine D2 receptors

DA: Dopamine agonist

MGMT: O6-methylguanine DNA methyltransferase

TMZ: Temozolomide

VEGF: Vascular endothelial growth factor

PRL: Prolactin

GH: Growth hormone

ACTH: Adrenocorticotropic hormone

TSH: Thyroid stimulating hormone

FSH: Follicle-stimulating hormone

NF: Non-functioning

## Competing interests

The authors declare that they have no competing of interests.

## Authors’ contributions

YW, JL and CM designed the research; YW, JL, YH, MT, SW, WL and ZL performed the research; WL and ZL evaluated the pathological sections and scored the extent of staining; JL and YW analyzed the data; JL, YW and CM wrote the paper, CM revised the paper. All authors read and approved the final manuscript.
